# Supplementary material for: Are carbon emissions trading and green financial instruments synergistic? -Comprehensive quantitative research based on content analysis
Source: PLoS One. 2024 Mar 7;19(3):e0298601. doi: 10.1371/journal.pone.0298601 (PMC10919601; doi:10.1371/journal.pone.0298601)
Supplement: S1 Data — (ZIP) [file pone.0298601.s001.zip › Data/地级市Z_F工作报告.zip!/╨┬╜«/╬┌┬│─╛╞δ╩╨/└┤╫╘╣1⁄2╓┌║┼í╛┬φ┐╦╩2╛▌═°í┐_2017.docx]

各位代表：

现在，我代表市十五届人民政府，向大会报告工作，请予审议，并请各位政协委员、列席代表提出意见。

市十五届人民政府工作回顾和2016年工作

过去的五年，是全市上下克难攻坚、奋力开创首府改革发展稳定新局面的五年，是在全面建成小康社会、实现社会稳定和长治久安伟大征程中阔步前进的五年。在自治区党委、自治区人民政府和市委的坚强领导下，在市人大、市政协的监督支持下，我们以党的十八大和第二次中央新疆工作座谈会精神为指引，坚定不移贯彻以习近平同志为核心的党中央治疆方略，紧紧围绕社会稳定和长治久安总目标，全面落实自治区党委、自治区人民政府和市委的重大决策部署，解放思想、开拓创新、奋力拼搏，圆满完成了市十五届人大确定的各项目标任务，首府经济社会发展取得了瞩目成绩，实现了“十三五”良好开局。

——五年来，我们着力持续完善维稳工作机制、强化维稳工作措施，稳定的基础不断夯实。牢固树立社会稳定和长治久安总目标，坚持主动进攻、先发制敌，严打暴 恐斗争成效明显。持续加强以人员密集场所为主的安全防范，强化以流动人口为主的社会管理。持续加强以社区为主的基层阵地建设，社区条件显著改善。深入开展民族团结宣传教育，依法加强宗教事务管理，“去极端化”工作扎实推进。广泛发动各族群众参与综治维稳和平安建设，扎实开展“访惠聚”活动，全市稳定的基础不断巩固。

——五年来，我们着力转方式调结构促发展，经济实现持续健康发展。主动适应经济发展新常态，紧贴民生、紧抓机遇加快发展，为首府转方式调结构、保持经济持续健康发展提供了强大动力。2016年主要经济指标较2011年均实现大幅增长，其中地区生产总值由1520亿元增长到2459亿元（按可比口径计算），年均增长12.1%；一般公共预算收入由206.2亿元增长到369.7亿元，年均增长12.4%；固定资产投资由635.1亿元增长到1607亿元，年均增长20.4%；社会消费品零售总额由695亿元增长到1238亿元，年均增长12.2%；全口径外贸进出口总额由569亿元增长到873亿元，年均增长8.9%。经济转型升级步伐加快，科技创新能力持续提升，三个国家级开发区引领作用增强，先进装备制造业、战略性新兴产业、高新技术产业快速发展，现代服务业比重大幅上升，具有首府特色的现代产业体系基本形成。

——五年来，我们着力推进创新驱动战略，改革开放取得重大突破。完成新一轮机构改革任务，两个国家级开发区与新市区、头屯河区“区政合一”深入推进，区域融合与兵地融合取得新进展。中心城区“大建委”体制不断完善，街道社区管理体制改革不断突破。实行规划土地市级统一管理，率先公布权责清单，下放市级管理权限515项，市级行政审批项目缩减50%以上。深入实施注册登记制度改革，多元化投融资格局基本形成。国有资产监管体系日趋完善，国企改革不断深化，国有经济活力进一步增强，财税、工商和社会领域改革均取得重要进展。积极融入丝绸之路经济带核心区建设，“五大中心”建设加快推进，成功举办五届中国—亚欧博览会，发起并举办四届丝绸之路经济带城市合作发展论坛。对外交流日益频繁，引进区外到位资金2966亿元，实际利用外资78亿元，乌鲁木齐区域影响力大幅提升。

——五年来，我们着力抓好民生工程建设，各族群众生产生活条件持续改善。每年办好100件以上民生实事，市财政用于民生领域支出达1394亿元，占同期一般公共预算支出的72.7%。坚持就业第一，累计新增城镇就业48.43万人，城镇登记失业率始终控制在3.7%以内。城镇居民人均可支配收入和农村居民人均可支配收入分别达到34200元和16400元，增长1.12倍和94.4%。建成540个肉菜副食品直销点，累计投放储备肉菜19.2万吨，实现稳价惠民；实施大规模棚户区和老旧小区改造，新建棚改房、保障房和农村“两居”住房15.15万套；建成国内一流的快速公交BRT系统7条线120公里，城乡公交网络体系日益完善。义务教育阶段学校标准化建设、均衡化发展任务全面完成，各类教育教学水平稳居全疆第一。在全疆率先开展新农合与城镇居民基本医疗保险并轨，基本药物制度全覆盖，实现了人人享有基本公共卫生服务目标。社会养老、福利、救助、慈善等事业取得长足发展，社保覆盖面不断扩大、保障水平不断提升。文化、广播电视等服务体系日益完善，各族群众精神文化需求得到有效保障。在全疆率先完成脱贫攻坚任务，2015年全面建成小康社会总体指标实现程度达到97%，荣获“全国十大幸福城市”称号。

——五年来，我们着力抓好城乡规划建设管理，城乡面貌明显改观。投入2000多亿元用于城市建设，城市环境和品质持续优化提升。“田”字路高架、东绕城高速、兰新高铁及乌鲁木齐站等重大工程相继建成，地铁、城际铁路、国际机场改扩建等重大项目加快实施，现代综合交通枢纽日臻完善。高铁、会展等新区基本建成，各类大型城市综合体和现代居住小区大量涌现，五年拓展城市空间200多平方公里。实施“煤改气”工程，我市成为全国首个气化城市，大气污染治理取得历史性突破。持续开展环境综合整治，生态治理保护深入推进，新增绿化面积23.32万亩，新建小游园小绿地小水面664个，城市绿化覆盖率达40.5%。超额完成污染物总量减排任务，万元GDP能耗大幅下降。成功创建国家园林城市、节水型城市和节能减排财政政策综合示范城市。

2016年是“十三五”规划的开局之年。我们认真贯彻落实市委十届十四次全委（扩大）会议和市第十一次党代会精神，紧紧围绕社会稳定和长治久安总目标，坚持稳中求进、改革创新，圆满完成了市十五届人大五次会议确定的目标任务，全市经济社会保持了持续稳定发展。

一、稳定基础日益牢固

一是严打严防措施不断强化。扎实开展严打专项行动，高标准规划建设便民警务站，强化流动人口和出租房屋精细化管理。扎实开展“访惠聚”活动，持续堵塞漏洞、消除隐患，社会稳定的基层基础不断巩固。

二是民族团结氛围日益浓厚。大力开展民族团结进步年活动，坚持不懈加强民族团结宣传教育。推进各民族 嵌 入式小区示范工程，掀起“民族团结一家亲”活动热潮，促进了各族群众相互尊重、相互学习、相互帮助。

三是宗教工作深入推进。依法加强宗教事务管理。充分发挥爱国宗教人士和信教群众作用，规范解经讲经，引导信教群众抵制极端。依法治理“三非”活动，坚决取缔地下讲经学经活动，有效遏制了宗教极端思想传播。

二、经济保持稳定增长

预计全年地区生产总值增长7.6%；固定资产投资总额与上年持平；社会消费品零售总额增长7.5%；全口径外贸进出口总额增长5%；一般公共预算收入增长0.27%；城镇居民人均可支配收入增长8.2%；农村居民人均可支配收入增长9.2%；居民消费价格指数101.5%。

一是“五大任务”落实成效明显。制定“两方案一通知”“12方面110条”等措施，完成63万吨煤炭产能退出任务，关停4家自备电厂，淘汰8家落后产能企业。实施契税财政补贴等优惠政策，房地产去库存工作稳步推进。加强政府债务管理，积极防范金融风险。继续执行工商用电同价，推进落实直接交易电价政策。积极落实“营改增”等结构性减税、普遍性降费和降低社会保险费率政策，有效降低企业经营成本。行政审批、国资国企、能源资源、财税金融等九大行业领域薄弱环节不断完善和加强，经济社会发展短板得到有效弥补。

二是工业转型升级步伐加快。高新技术产业实现增加值131.7亿元，增长3%；战略性新兴产业实现增加值125.7亿元，增长3.4%。铁建重工高端装备基地、神华新疆年产68万吨煤基新材料、莱沃科技等重点项目建成投产，中国中车、广汽乘用车、金昇智能机械等一批先进装备制造业项目开工建设。“两化”融合快速推进，建成国家级“两化”融合示范企业7家、自治区级示范企业及“两化”融合支撑单位73家。智慧乌鲁木齐建设全面推进，天山云乌鲁木齐云计算产业基地项目开工，曙光云计算中心正式投用。

三是现代服务业加快发展。电子商务、软件信息、互联网金融等新兴业态加速集聚，新疆软件园入驻企业220余家。建成国家、自治区电子商务示范基地3个、示范企业26家，“惠民网”签约超市8500余家。互联网金融中心交易量突破40亿元，占全疆90%。举办各类展会132场，增长17.9%。新增特色美食街（区）5条。检验检测认证产业园开工建设，纺织品服装商贸中心、服务外包基地等项目加快建设，获批中国服务外包示范城市。旅游业提档升级步伐加快，丝绸之路经济带旅游集散中心主体完工，南山国际旅游区核心区、丝绸之路欢乐大世界等一批旅游项目加快建设。全年接待游客2533万人次，增长15%；实现旅游总收入340亿元，增长14%。

四是农业农村工作取得新进展。播种各类农作物67万亩，完成设施农业提质增效5000亩。创建“一村一品”示范村16个。培育各级规范化合作社201家，认定家庭农场18家。建设安居富民住房10520户，启动10个新农村示范村创建工作，建成农村公路95公里，实施了一批供排水、污水处理、园林绿化、环境卫生等基础设施配套项目，农业农村发展水平进一步提高。

五是科技创新能力进一步增强。组织实施127项重点产业关键技术研究和成果转化项目，新增高新技术企业45家、创新型（试点）企业44家，新建工程技术研究中心（重点实验室）6家。丝绸之路经济带创新驱动发展试验区、国家技术转移东部中心新疆分中心等项目落地建设。实施“红山众创行动计划”，深入推进“双创”工作，获得国家“双创示范城市”奖补资金2亿元。高新区（新市区）、经开区（头屯河区）成功创建首批全国科普示范区。

三、改革开放全面深化

一是重点领域改革深入推进。国资国企改革、属地注册改革、商事制度改革取得积极进展。行政审批制度改革步伐加快，市政务服务中心建成启用，在全疆率先公布市级部门责任清单，确立了3265项职权事项。大力推广政府和社会资本合作模式，签约13个PPP项目，投资总规模达773亿元。企业上市工作取得新进展，2家企业成功上市，6家企业成功登陆“新三板”。城管体制改革全面启动，人才管理和出租车行业体制改革全面深化。

二是对内对外开放进一步扩大。综合保税区顺利通过国家验收，国际贸易服务区启动建设。多式联运海关监管中心建成使用，国际陆港区与国内外多个主要海陆港、铁路港实现战略合作。铁路西站口岸、机场进境免税店成功获批，国际快件监管中心建成启用。西行国际货运班列实现常态化运行，面向中欧、中亚班列乌鲁木齐集结中心初步形成。跨境电子商务综合试验区申报工作加快推进。首家外国银行—巴基斯坦哈比银行落户我市。以国际医院、友爱医院为标志的丝绸之路经济带核心区医疗服务中心建设加快推进，成功举办一系列丝绸之路经济带健康论坛，签署国际国内各类医疗合作协议51项，跨境远程医疗服务平台建成启用，跨境云医院集群建设初具规模。

四、民生改善取得新成效

一是就业和社会保障工作成效显著。新增城镇就业11.16万人，城镇登记失业率控制在3%。实施大学生创业扶持计划，423名大学生实现创业。帮扶困难群体就业6642人，农村富余劳动力转移就业6991人。完成各类职业培训12.21万人。实施全民参保计划，社会保险覆盖面持续扩大，五项社会保险净增12.65万人。全面启动机关事业单位养老保险制度改革，建立城镇职工基本医疗保险门诊统筹制度和城镇职工大病保险制度，与8省市实现异地就医结算。连续第12年调整企业退休人员养老金，人均月养老金提高到2839元。城乡居民最低生活保障标准分别提高到每人每月410元和240元。

二是教育、卫生、体育等各项社会事业加快发展。投入53.74亿元，新（改）建中小学校21所、城乡学校少年宫64个。市青少年综合实践教育中心投入使用。优质资源北扩工程加快推进，市第130中学招生办学、第1中学新校区主体完工。城乡教师交流活动有序推进，边远薄弱学校师资力量不断加强。落实各类城乡义务教育保障资金4.29亿元，发放普惠性、公益性民办幼儿园奖补资金3482万元，新建公办幼儿园5所，实现农村适龄儿童免费入园。市属3家医院通过三甲医院评审，分级诊疗和医师多点执业稳步推进，优质医疗资源加快向基层流转。全面启动全民健康工程，提前完成第一轮全民体检工作。基本公共卫生服务均等化水平稳步提高，免费向城乡居民提供15类51项基本公共卫生服务，人均补助标准达到85元。圆满完成全国第十三届冬季运动会乌鲁木齐赛区的赛事组织和服务保障工作，我市代表队取得12金9银12铜的好成绩。启动乌鲁木齐奥林匹克体育中心项目建设。人口和计划生育服务管理不断加强，人口出生率为9.86‰。食品药品专项整治扎实开展，群众饮食用药安全得到保障。安全生产应急管理能力显著提升，各类生产安全事故下降8.32%。新闻出版、防灾减灾、质监、气象、地震、地方志、档案等工作取得新进展，妇女儿童、残疾人和红十字事业取得新成绩。

三是文化建设取得新进展。大力开展精神文明创建活动，深入推进社会主义核心价值观和中国梦宣传教育。“文明承诺”“文明接力”“文明交通出行”等活动成效显著，学雷锋社会志愿服务注册人数达29.7万人，市民文明素质明显提升。顺利通过中央文明委第二年度全国文明城市创建检查验收，荣膺全国双拥模范城“八连冠”。深入开展文化惠民活动，政府购买文化惠民演出280场次。开展“我们的中国梦”文化进万家惠民活动520余场次、百日广场文化活动3500余场次，放映公益电影1.13万场次。市文化中心及区（县）文化馆、图书馆等基层文化基础设施建设快速推进。新增自治区级（市级）文化产业示范基地29个、特色文化街区4条。我市在第十二届深圳文博会签约28.9亿元，增长30.1%。

四是百件民生实事全面完成。新建13个社区蔬菜副食品直销点。投入65亿元实施棚户区改造项目22个、建设房屋10690套。回购900套商品房和经济适用房作为公租房，降低保障房申请门槛，4000户中低收入家庭圆了“住房梦”。建成BRT6号线支线，新增和优化公交线路30条，试点开通定制公交线路2条。落实残疾人免费乘坐公交车政策。新设立养老机构3个，新增养老床位307张。建成“儿童之家”19个，符合条件的困境儿童全部纳入城乡低保。

五、城市规划建设管理和生态建设再上新水平

一是城乡规划进一步完善。城市总体规划2016年修改版获国务院批复。完成“五大中心”专项规划，组织开展近期建设规划及老城区改造提升规划设计方案。开展会展片区提升、城市风貌特色建设、城市山水风貌保护等详细规划和城市设计。完成综合管廊、海绵城市、慢行系统等专项规划。

二是基础设施建设取得新进展。国际机场改扩建征收工作加快推进。地铁1号线14座车站封顶，贯通区间17.3公里，首列电动客车接车进场；2号线（一期）建设加快推进，3、4号线（一期）开工建设，5、6号线启动前期工作。珠江路立交改造、九家湾立交改造、苏州路东延、三屯碑路西延等项目建成通车，疏通了城市重要交通节点。国道216线提升改造、喀什路东延、新医路西延等道路建设项目快速推进，城市主干路网体系不断完善。地下综合管廊等重点基础设施建设加快推进，城镇供水、供热、供气和污水、垃圾处理等基础设施服务能力显著提升。

三是老城区改造和新区建设加快推进。按照“一年开工、两年见效、三年完善”的要求，制定老城区改造提升规划实施意见、工作方案和征收补偿安置工作导则，全面启动并加快推进老城区改造提升工作。加快构建“多中心、组团式”城市发展格局，高铁、会展、白鸟湖、高新区北区等新区公共设施更趋完善，城南经贸合作区、古牧地新区建设加快推进。

四是城市管理水平进一步提高。持续开展环境综合整治，规范外立面色彩，粉刷沿街建筑1400余栋、146万平方米，整治背街小巷115条，创建标准化示范街8条，打造景观照明示范街5条。城市出入口及城乡结合部重点整治初见成效。拆除违法建筑101.7万平方米。建立“以奖代补、以奖促治”的城市运行管理综合考核体系，城市管理服务水平进一步提升。

五是环保和生态建设持续加强。不断巩固大气污染治理成果，深化工业企业污染治理，完成重点企业脱硫、脱硝配套污染防治设施及封闭料仓建设。完成加油站、储油库油气回收治理改造191座。改造换热站116座、老旧供热管网190公里。实施既有建筑供热计量及节能改造506万平方米，新增电采暖面积92万平方米，完成燃气锅炉烟气余热回收改造1715蒸吨。淘汰“黄标车”及老旧车辆1.12万辆。深化排污权有偿使用交易试点，减排二氧化硫3740吨、氮氧化物7758吨。全年空气优良天数达246天，优良率达67.2%，环境空气质量持续改善。新增绿化面积3万亩，完成森林抚育2万亩。新增街旁绿地、小游园100个，实施荒山绿化改造提升公园项目8个。一号冰川、柴窝堡湖等重点水源涵养区生态修复治理工作持续加强。大浦沟生活垃圾填埋场关停，大黑沟垃圾综合处理场投入运营。严格落实取水许可和水资源论证制度，合理配置有限水资源。

六、政府自身建设迈上新台阶

坚定正确政治方向，严守政治纪律和政治规矩，扎实开展“两学一做”学习教育。切实推进法治政府、创新政府、廉洁政府和服务型政府建设，各级政府服务能力和水平显著提高。加强政风行风建设，坚持不懈落实中央八项规定、自治区党委和市委十条规定，坚决纠正“四风”，实施作风建设“三项治理”，干部作风持续改善。深入开展法治宣传教育，获得全国法治宣传教育先进城市称号。依法治市工作不断深化，编制完成“十三五”依法治市规划纲要和“七五”普法规划，实施修订政府规章5件。自觉接受市人大及其常委会的依法监督和市政协的民主监督，积极听取各民主党派、工商联、无党派人士和人民团体意见，办理自治区、市人大代表议案、建议和政协提案447件，办复率100%。政务公开和网络问政工作不断加强，信访和行政调解工作机制进一步完善。扎实开展人民调解化解矛盾纠纷工作，调解成功率达99%。行政监察、审计监督工作扎实推进，严肃查处一批贪污受贿、损害群众利益的违法违纪案件，预防和惩治腐败取得新成效。

各位代表，回顾本届政府的工作，我们深深体会到：做好乌鲁木齐工作，必须牢固树立政治意识、大局意识、核心意识、看齐意识，始终如一地向以习近平同志为核心的党中央看齐，在政治上、思想上、行动上同以习近平同志为核心的党中央保持高度一致，坚定坚决地落实自治区党委和市委各项决策部署；必须全面贯彻落实社会稳定和长治久安这一总目标，各项事业都要服从服务于总目标，所有工作都要以此为着眼点着力点；必须看到发展的后发优势，注重发挥比较优势，深入挖掘潜在优势，积极转化相对劣势，加快转方式调结构促增长，发展培育多元支柱产业，不断夯实经济发展的产业基础；必须始终坚持创新的理念，深化改革和扩大开放，激发内在活力，以宽广的视野谋划发展、推动发展；必须立足发挥中心带动作用，同步推进老城区改造提升和新区建设，拓展城市空间和完善城市功能；必须坚持一切为了人民、一切依靠人民，想方设法保障和改善民生，以为民服务的实效赢得民心，让各族群众过上更加幸福安定的生活；必须坚持不懈地转作风、促落实，把提高行政效能作为根本保障，加强制度建设和流程再造，提高政府的执行力、创新力、公信力和约束力。

各位代表，回顾本届政府的工作，我们深深感到：所有这一切，都是自治区党委、自治区人民政府和市委正确领导的结果，是全市各族人民共同努力和各方面大力帮助支持的结果。在此，我代表市人民政府，向全市广大工人、农民、干部和知识分子、兵团职工，向各民主党派、工商联、无党派人士，向人民团体和社会各界人士，向驻乌人民解放军和武警部队指战员、公安干警，向中央驻乌单位、所有援乌干部，向人大代表、政协委员表示衷心的感谢并致以崇高的敬意！

在肯定成绩的同时，我们也清醒地看到工作中还存在着问题和不足：一是对反分裂斗争长期性复杂性尖锐性的认识需要不断强化，维护稳定能力有待进一步提升，长治久安深层次问题亟待有效解决；二是产业发展层次总体偏低，实体经济支撑力不足，创新驱动能力较弱，保持经济快速增长的难度加大；三是对外开放深度广度不够，城市国际化水平不高，基础设施建设相对滞后；四是优质公共服务供给不足，城乡一体化步伐缓慢，改善民生离各族群众期望还有差距；五是大气污染防治工作任重道远，区域联防联控难度较大，人居生态环境有待进一步改善；六是政府作风建设还需进一步加强，服务群众的能力仍需提高。对此，我们将在今后的工作中采取有效措施，认真加以解决。

今后五年目标任务和2017年主要工作

今后五年，是我们深入贯彻落实以习近平同志为核心的党中央治疆方略，实现社会稳定和长治久安总目标的关键时期，也是我们持续推进稳定与发展，全面建成更高水平小康社会，加快建设现代化国际城市的重要阶段。

今后五年政府工作的指导思想是：高举中国特色社会主义伟大旗帜，深入贯彻第二次中央新疆工作座谈会和自治区第九次党代会精神，树牢社会稳定和长治久安总目标，全面落实自治区党委决策部署，坚定市第十一次党代会确定的奋斗目标和工作思路不动摇，统筹推进“五位一体”总体布局，协调推进“四个全面”战略布局，牢固树立和贯彻落实新发展理念，积极融入自治区丝绸之路经济带核心区建设，坚定不移维护社会大局稳定，坚定不移保障和改善民生，坚定不移推动经济持续健康快速发展，坚定不移推进现代化国际城市建设，坚定不移促进民族团结宗教和谐，加快打造和谐稳定、既优又强、民族融合、宗教和睦、独具魅力、干净美丽的首府，努力把乌鲁木齐建设成为西北地区特色鲜明、绿色健康、宜居和谐的现代化国际城市。

今后五年我市经济社会发展的主要目标是：到2021年地区生产总值达到4000亿元左右，年均增长7.5%左右。固定资产投资年均实现两位数增长；社会消费品零售总额年均增长8%左右；一般公共预算收入年均增长5%左右；全口径外贸进出口总额年均增长5%左右。城镇居民人均可支配收入年均增长8%左右，农村居民人均可支配收入年均增长9%左右。

为实现上述目标，我们将重点把握好以下六个方面：

一是加快打造和谐稳定首府。深刻认识社会稳定和长治久安总目标的重大意义、基本要求和主要举措，坚持把总目标作为统领首府各项工作的总纲，所有工作都要服从服务于总目标，围绕总目标来开展、来谋划、来推动。认真贯彻自治区党委出台的各项维稳措施，打好维护稳定“组合拳”，做到“六个抓好”“五个管住”“四个全覆盖”，筑牢维护稳定的坚强防线和铜墙铁壁，确保社会大局稳定。

二是加快打造既优又强首府。以推进供给侧结构性改革为主线，深入推进“三去一降一补”五大任务，全力稳增长、促改革、调结构、惠民生、补短板、防风险，争当全疆改革发展排头兵。坚持“一产上水平、二产抓重点、三产大发展”，着力打造商贸物流、现代金融、先进装备制造、智慧安防、化工、新材料六个千亿级产业集群，文化旅游、信息、特色餐饮、新能源、纺织服装、健康六个百亿级产业集群，加快特色农业产业集群建设，加快构建主业突出、特色鲜明、竞争力强的现代产业体系。强化创新驱动发展，推动大众创业、万众创新，力争科技研发投入占GDP比重达2.5%、高新技术产业总产值翻一番。加快丝绸之路经济带核心区“五大中心”建设，强化首府交通枢纽、商贸物流、金融、文化科教、医疗服务功能，着力构建全方位开放新格局，提升首府对外影响力辐射力。以争取人心、赢得民心为目标，全力落实安全、就业、教育、医疗、社保、扶贫、安居、暖心、文化“九大惠民工程”，千方百计增加公共服务和社会福利，让各族群众有更多的参与感、获得感和幸福感。

三是加快打造民族融合首府。高举各民族大团结旗帜，坚持中国特色解决民族问题的正确道路，大力促进各民族共同团结奋斗、共同繁荣发展，努力打造民族团结之城，力争用3年时间创建成全国民族团结进步示范市。坚持把加强民族团结纳入国民教育、干部教育、社会教育，引导各族干部群众牢固树立“三个离不开”思想，增强“五个认同”，促进各民族和睦相处、和衷共济、和谐发展。加快构建各民族相互嵌入式的社会结构和社区环境，广泛开展民族团结进步创建、“民族团结一家亲”等活动，促进各民族交往交流交融。

四是加快打造宗教和睦首府。坚定不移贯彻党的民族宗教政策，坚持保护合法、制止非法、遏制极端、抵御渗透、打击犯罪的原则，依法加强宗教事务管理，抓好“五好”宗教活动场所、“五好”宗教人士创建活动，发挥好爱国宗教人士和信教群众在社会稳定和长治久安中的作用，积极引导宗教与社会主义社会相适应。加强清真寺民主管理组织建设和干部住寺工作，完善管理运行服务模式，实现清真寺管理服务全覆盖。

五是加快打造独具魅力首府。坚持“三区七组团”发展格局，加快“南控北扩、西延东进”步伐，全面推进老城区改造提升和新区建设工作，完善现代化国际城市框架。加快推进以地铁为骨干的现代综合交通枢纽建设，确保“十三五”期间地铁1号线、2号线（一期）建成运营。加强建筑特色风貌建设、山水特色风貌保护，精心做好城市“水文章”，系统规划打造水系景观。创新城市管理服务体系，提高精细化管理水平，推广电子政务服务、城市智能管理和智慧民生服务，基本建成智慧城市。

六是加快打造干净美丽首府。大力推进生态建设和环境保护，着力构建“四区、四廊、一环”的生态保护空间格局，努力让首府天蓝地绿水清。打好大气污染治理攻坚战，强力推进大气污染联防联控，持续改善首府环境空气质量。加快城市“双修”工作，推进重大生态修复工程，加快绕城生态圈防护绿带建设，构建首府绿色生态屏障。促进绿色循环低碳发展，扩大清洁能源应用覆盖范围，推进全社会节能减排。积极申办中国国际园林博览会。实现创建国家生态园林城市、卫生城市、环保模范城市和全国文明城市目标。

2017年，是新一届政府的开局之年，做好各项工作意义重大。全市经济社会发展的主要目标是：地区生产总值增长7.5%，固定资产投资增长50%，社会消费品零售总额增长8%，全口径外贸进出口总额增长5%，一般公共预算收入增长5%，城镇居民人均可支配收入增长8%，农村居民人均可支配收入增长9%，城镇登记失业率控制在3.7%以内，居民消费价格涨幅控制在3.5%左右。

围绕这一目标，我们将重点做好以下几个方面工作：

一、全面落实各项维稳措施，筑牢社会稳定和长治久安根基

稳定是发展的前提基础，没有稳定一切都无从谈起。坚决落实自治区党委维稳“组合拳”，构建维稳常态，为全市经济健康快速发展创造良好环境。

一是严打严防保稳定。继续保持严打高压态势，坚持主动出击，毫不手软，坚决遏制“三 股 势 力”。全面落实社会面安全防控措施。进一步完善便民警务站运行机制，开展全天候全时段的警务巡控和便民服务。深入推进反分裂斗争教育，强化意识形态领域阵地建设，加强互联网管理和敏感舆论管控。积极打造“智慧安防”首府，大幅提升社会治理体系和治理能力现代化水平。

二是加强基层基础建设。推行社区阵地标准化建设，进一步充实社区工作者队伍，完善社区警务管理模式，加强群防群治和巡逻队伍建设，健全考核奖惩和激励保障机制，不断激发社区工作活力，提升维稳工作能力和服务管理水平。加强流动人口精细化服务管理和出租房屋分类管理，让流动人口更好融入社区生活，增强归属感和责任感。深化“访惠聚”工作，让各族群众更多享受发展成果，切实感受到党和政府的关怀，形成共同建设美丽首府的强大力量，筑牢社会稳定和长治久安的钢铁长城。

三是巩固民族团结良好局面。进一步深化“两模范”创建工作，全面掀起民族团结热潮。深入开展“民族团结一家亲”活动，促进各族干部群众结对子、认亲戚，勤走访、常团聚，互学语言，关爱帮扶，多层次多形式交往互动。推动建立各民族相互嵌入式的社会结构和社区环境，促进各民族交往交流交融，使各族群众学习在一起、生活在一起、工作在一起，形成你中有我、我中有你、密不可分的大好局面，让各族群众像石榴籽一样紧紧抱在一起！

四是促进宗教和睦和谐。进一步创新清真寺和宗教活动教育管理服务工作，确保宗教领域和谐稳定、宗教活动规范有序，营造平等团结、遵纪守法、爱国感恩的良好氛围。继续深化“双五好”创建活动。扎实推进“去极端化”工作，加大“三非”治理力度，大力批驳宗教极端思想，引导各族群众擦亮眼睛、树立正信、抵制极端。充分发挥伊斯兰教协会和爱国宗教人士作用，引导信教群众确立正道正信，与宗教极端势力作坚决斗争。

二、围绕构建现代产业体系，推动经济提质增效升级

实体经济是经济发展的根基。加大产业、基础设施等项目建设力度，加快三次产业发展，着力振兴实体经济，引领带动全市经济持续健康发展。

一是加大项目建设和投融资力度。全力推进2400亿元重点项目建设，迅速启动和实施一批重点产业、民生、基础设施建设等重大项目，力争在短期内形成对经济增长的拉动效应。紧紧围绕壮大产业集群和补齐发展短板，谋划一批新项目、好项目，积极落实项目建设条件，确保项目顺利实施。加大融资和资金争取工作力度，积极落实与各金融机构达成的3600亿元战略合作协议，确保完成全年融资1200亿元的目标任务。坚持“招大引强”和“招商选资”，吸引更多国内外企业特别是跨国公司进驻投资。在降低准入门槛、引导市场预期、增强企业投资意愿上下功夫，加强政策资源集聚和服务协同，根据企业类别精准施策，推动小微企业向“多、精、优”升级，鼓励骨干企业做大做强，调动民营企业积极性，扩大社会投资规模。

二是做强做优现代服务业。围绕打造千亿级商贸物流产业集群，深入推进传统商贸流通业升级改造，促进电子商务、连锁便利等新业态发展，鼓励商贸企业培育自有品牌、引进知名品牌，满足各族群众消费需求。加快国际陆港区、空港物流区等重大项目和城南经贸合作区建设，加大物流资源整合力度，加快物流设施现代化，推进城市共同配送。围绕打造千亿级现代金融产业集群，积极吸引各类金融机构入驻，推动科技金融创新中心等重大金融产业项目建设，促进金融产品和服务创新。围绕打造百亿级文化旅游产业集群，推进文化旅游深度融合，突出全域旅游发展理念，推动特色旅游项目建设，扶持培育旅游龙头企业，着力打造一批精品景区、特色旅游乡镇（村）。按照夏季搞活、冬季搞火、四季发展的要求，加快南山国际旅游区核心区改造提升，大力发展冰雪产业和现代马产业，推进丝绸之路经济带旅游集散中心、丝绸之路欢乐大世界、达坂城西部歌城小镇等项目建设，启动冰雪产业园建设，高水平办好丝绸之路冬夏两季冰雪风情节。加快推进乌鲁木齐县康养产业试点工作，抓好总投资500亿元1700公里旅游道路建设。力争全年接待游客2786万人次，增长10%，实现旅游总收入374亿元，增长10%。围绕打造百亿级信息产业集群，加快信息产业及软件等衍生产业发展，推进新疆软件园、国家电子商务基地、“一带一路”大数据分析中心、亚欧大数据交易中心等项目建设，启动物联网产业园建设，加快“三网融合”和智慧城市建设步伐。围绕打造百亿级特色餐饮产业集群，加快实施中央厨房、冷链配送等项目建设，抓好美食街区改造提升，引导特色餐饮企业走集团化、连锁化道路。推进会展业向品牌化、国际化发展，办好丝绸之路国际食品展交会、安防博览会等重点展会，力争全年办展150个以上，增长18%。

三是推动工业经济转型跨越发展。围绕打造千亿级先进装备制造产业集群，全力推动重汽、广汽、中车、金昇等项目建成投产，鼓励零部件企业与总装企业同步发展，力争关键零部件配套企业落户我市，填补和延展化工、汽车、工程机械、轨道交通等产业配套链条。围绕打造千亿级智慧安防产业集群，依托智慧安防产业园建设，重点在安防视频监控设备、安防设备制造等领域率先突破。围绕打造千亿级化工产业集群，加大“两化”融合力度，推动传统支柱产业技术改造、转型升级，加快建设石油化工集群、绿色煤化工集群和先进氯碱化工集群。围绕打造千亿级新材料产业集群，以众和股份、新特能源、紫晶光电等企业为龙头，做大做强铝基、硅基等新材料以及高分子材料、高性能复合材料等产业。围绕打造百亿级新能源产业集群，加快开展发电核心技术、关键材料和设备研究，推进达坂城区氢能源开发应用试点，继续扩大新能源产品的推广应用范围和规模。围绕打造百亿级纺织服装产业集群，加快乌鲁木齐国际纺织品服装商贸中心建设，积极推动纺织服装产品交易和集散平台发展，促进纺织服装产业升级。围绕打造百亿级健康产业集群，充分利用高新区（新市区）生物医药和健康产业优势，加快地方特色药研制开发和新型药物产业化进程，构建生物医药、生物制造产业链。

四是推进都市现代精致农业发展。围绕打造特色农业产业集群，加快农业产业化进程，积极培育现代农业园区。积极扶持特色种养业发展，推进设施农业提质增效，打造“米袋子”“菜篮子”“肉案子”工程。健全现代畜禽良种和蔬菜育苗繁育体系，加强农产品流通设施和市场建设，提高农业综合生产能力和效益。开发农业生态观光旅游、休闲康养体验功能，积极推进高新区（新市区）北区特色小镇和万亩水塘乡村采摘游、米东都市休闲农业区、达坂城生态观光旅游区发展。扎实推进新农村建设，打造一批旅游文化、特色养殖示范村、示范乡，建设环境优美、和谐宜居、具有首府特色的社会主义新农村。

五是强化创新驱动发展。增强科技创新的支撑引领作用，鼓励技术引进，加快提升关键核心技术攻关能力，开展促进企业科技成果转化行动计划，推动创新链和产业链融合发展。实施新兴产业培育壮大工程，推动配套延伸，为产业集群的格局重构提供强力支撑。推动丝绸之路经济带创新驱动发展试验区、小微企业创业创新示范基地建设，深入推进“红山众创行动计划”，推进知识产权保护工程，办好“第三届创新创业大赛”，加快打造创新型城市。鼓励设立国家级工程技术研究中心、院士工作站、博士后工作站等研究机构，组建市级工程技术研究中心（重点实验室）6家，新增高新技术企业和创新型（试点）企业30家。加快引进各类专业技术人才，推进职称互通互认，促进人才合理流动和科学配置。

三、全面深化改革开放，争当全疆改革发展排头兵

“改革开放只有进行时没有完成时”。聚焦党中央和自治区党委全面深化改革重大部署，坚持以改革破解突出矛盾、以开放赢得发展先机，努力建成丝绸之路经济带核心区支撑点、桥头堡。

一是加快重点领域改革步伐。坚持以深化供给侧结构性改革为主线，抓好“三去一降一补”五大任务落实。坚持“加减乘除法”并举，积极化解煤炭产业过剩产能。着力规范房地产市场秩序，激活市场有效需求，减少商品房库存。有效降低企业杠杆率，防范金融领域风险。打好降成本“组合拳”，采取减税、降费、让利和优化服务等措施，推进补短板、添动能，不断提高实体经济竞争力。全面深化行政管理体制改革，完成新一轮市、区（县）两级政府机构改革任务，实现政府工作高效流畅运转。加快推进城管体制改革，构建市级统筹、区（县）为主、权责统一、精细管理的智慧城管新体系。加快行政审批制度改革，规范优化政务服务事项，加快推进“互联网+政务服务”，加大“放管服”改革力度，推进“一门式、一网式”政务服务模式改革，着力打造全疆营商示范窗口。加快三个国家级开发区改革，重点在管理体制、政策优势、人才创新上放手、放松、放宽，进一步擦亮国字号金字招牌，在首府创新发展中先行先试、探路领跑。深化国资国企改革，积极推动资源类央企属地注册，加快股权多元化、资产证券化步伐，推动国有企业主动参股央企和自治区企业，支持乌鲁木齐银行上市。扎实推动国企统一监管，完善行政事业单位国有资产集中统一管理工作机制，合理配置和有效利用国有资产。深化投融资体制、财税体制、商事制度、农村产权制度及重点领域价格改革。推进非公经济配套改革试验区试点，大力支持非公有制企业创业创新、转型升级，推动民营企业依法平等参与市场活动，促进非公有制经济快速发展。

二是以“五大中心”建设为重点，全方位扩大对外开放。落实好自治区推进丝绸之路经济带核心区建设实施意见和行动计划。加快集装箱中心站建设和北站物流资源整合，推进机场改扩建四期工程，加快跨境电子商务综合试验区申报建设，推进陆港、空港、海港、信息港等多港联动，全面打通境内外联通“动脉”。完善多式联运海关监管中心和综合保税区运行机制，提升中欧国际货运班列集结中心常态化运营水平。加快信息基础设施建设，积极参与连接亚欧非的西向国际通信、信息传输和光缆大通道建设。加强与国际性金融组织、周边国家金融机构的沟通交流，推动取得更多务实合作成果。优化提升跨境远程医疗服务平台功能，加快医疗旅游推介平台等一批重点项目建设，打造一批具有国际服务水平的重点医疗机构。加快推进亚欧商贸物流职业教育培训中心建设，办好第五届丝绸之路经济带城市合作发展论坛、首届丝绸之路卫生合作论坛，不断提升对外影响力辐射力。积极发展出口导向型经济，促进特色农产品、资源深加工、高新技术产品出口。加快推进跨境电子商务服务平台、“丝贸通”一站式外贸服务平台建设，促进外贸转型升级。以服务外包产业园和多语种服务平台等项目为重点，加快推进服务外包示范城市建设。鼓励支持企业“走出去”投资建厂办园区，加快我市企业境外园区建设。

四、深入落实九大惠民工程，大幅提升各族群众幸福指数

“人民对美好生活的向往，就是我们的奋斗目标”。要通过持续推进各项重大惠民工程，让各族群众不断增强获得感，让各族人民生活的更幸福、更和谐、更美好！

一是推进安全惠民。深入开展平安首府建设，实施“平安细胞”工程，平安区（县）创建率达到100%，优秀率达到50%以上，人民群众安全感和满意度达到90%以上。加强食品药品安全监管，积极推动国家食品安全示范城市创建工作。完善防灾减灾救灾体系，建立电梯应急救援平台。强化安全生产风险防控体系建设，有效防范各类事故发生。

二是推进就业惠民。坚持以产业带动就业、创业促进就业、政策扶持就业，创造更多就业机会。做好高校毕业生、就业困难人员等重点群体就业，实现城镇人员就业10万人，城镇登记失业率控制在3.7%以内。建设“一站式”就业和社会保障服务大厅60个，农村富余劳动力转移就业6000人，完成各类职业培训11万人。培育5个具有聚集效应的创业孵化基地，新增创业4000人，带动就业1.2万人。建立高校“就业创业指导服务中心”和“创业苗圃”，大中专应届毕业生就业率达到85%以上，扶持400名高校毕业生自主创业，企业新吸纳大中专毕业生8500人以上。

三是推进教育惠民。深入推进教育强区（县）工作，提高中小学标准化建设和均衡化发展水平，义务教育阶段实施教科书免费政策，高中阶段实施免费教育。全面普及农村学前三年免费双语教育，建设42所农村双语幼儿园，逐步实现全市学前免费教育。推动优质教育资源北扩和普通高中扩容提质，启动第130中学高中招生，推动第1中学新校区使用，筹建第20、23中学分校。启动市职业教育城（园区）建设，推动职业教育集团化办学、特色化建设、订单式培养。积极推进双语教育，完善特殊教育体系，支持和规范民办教育发展。

四是推进医疗惠民。深化医药卫生体制改革，实行医疗医保医药联动，推动分级诊疗和区域医疗联合体系建设。促进基本公共卫生服务均等化，年内全面完成乡镇卫生院和村卫生室标准化建设任务，加快社区卫生服务机构标准化建设。全面实施全民健康工程，每年为各族群众提供一次免费健康体检。着力发展特色专科医疗服务，高标准打造国际医院、友爱医院、儿童医院城北新院等一批面向全疆、辐射周边国家和地区的现代化医院。坚持计划生育基本国策，促进人口长期均衡发展。促进妇女儿童和红十字事业发展。

五是推进社保惠民。健全覆盖城乡的社会保障体系，大力推进全民参保计划，持续扩面提标，确保各险种征缴率达95%以上。实行大病保险和门诊统筹，减轻各族群众看病负担。推进智慧（电子）社保建设，扩大基本医疗保险异地就医结算范围，实现个人缴费网上办理。

六是推进扶贫惠民。进一步提高城乡低保标准，完善特困人员救助供养制度，实施鳏寡孤独、残疾人等特殊困难群体精准帮扶，兜住兜牢困难群众生活底线。继续加大对口扶贫工作力度，积极发展慈善事业。

七是推进安居惠民。以老城区改造提升为契机，加快惠及13万群众的保障性住房建设，完成5.2万户棚户区改造和5000户农牧民“两居”工程建设任务。完善住房保障体系，适度放宽住房保障申请条件，解决4000户中低收入家庭住房困难问题。推行物业企业分类评定、诚信管理等制度，提升物业市场管理水平。

八是推进暖心惠民。加快推进“医养结合”试点，完善养老服务设施，建成15个农村幸福互助院、社区日间照料中心。规划建设城市慢行系统，发展定制公交等新型服务业态，试点公交半小时优惠换乘。鼓励社会力量投资建设公共停车场，试点小区对外错峰停车和企事业单位对外开放停车场。加强国防教育，深入开展双拥共建活动，推进军民融合发展，落实优抚安置政策，提高重点优抚对象抚恤标准，争创全国双拥模范城“九连冠”，共同谱写军政军民双拥共建新篇章。

九是推进文化惠民。着力提升公共文化服务能力，完成市文化中心“六馆”主体工程，加快推进基层文化设施建设，进一步完善政府购买文化演出机制，将更多公共文体设施免费向群众开放。大力发展少数民族文化事业，鼓励创作更多反映少数民族现代文明生活的文艺精品。实施戏剧振兴、文学艺术提升、影视精品打造、网络文艺发展、文艺领军人才培育“五大工程”，全面丰富优质文化产品供给。积极培育城市特色文化，打造3个特色文化产业示范园、2个特色文化街区、10家市级文化产业示范基地。加快推进奥林匹克体育中心和城乡公共体育设施建设，办好市第四届运动会。广泛开展全民健身活动，加快促进体育产业发展。大力培育现代都市文明，不断提高市民文明素质和社会文明程度，全力争创全国文明城市。推动统计、外事、工商、质监、人防、气象、档案、新闻出版、史志编纂等各项社会事业蓬勃发展。

五、围绕建设现代化国际城市，大力提升城市功能品质

按照“尊重一个规律，坚持五个统筹”要求，高起点、高标准、高水平、大手笔推进城市规划建设管理，提升城市综合服务功能和辐射带动能力。

一是提升城市规划水平。发挥规划引领指导和调控作用，开展2030年新一轮城市总体规划修编，加强重点地段控规和城市设计，完善提升各类专项规划和控制性详规，做到控规全覆盖。推动以城市总体规划为龙头的“多规合一”，促进经济社会发展目标、城市空间坐标、土地利用指标等协调一致。提高城市设计水平，编制完成建筑特色风貌建设规划、山水特色风貌保护规划，控制和保护好城市天际线、山际线和水际线，精心精细精致管控城市建筑体量风格和整体布局，彰显民族特色、现代特色和宜居特色。

二是推进老城区改造和新区建设。把老城区改造提升作为“一号工程”，按照“一个调整、两个减少、三个提升、四个增加、五个结合”的思路，采取市场化运作方式，加快推进征收工作和安置新区建设，改造一批凸显城市特色的街区，完善老城区公共服务和基础设施，鼓励和引导多民族嵌入式居住，提升老城区整体品质与发展活力，让各族群众生活的更方便、更舒适、更美好。按照打造产城融合、宜业宜居现代化新城区的要求，加快推进会展、高铁、空港、白鸟湖、城北新区、国际陆港区、城南经贸合作区等新区建设，提升城市国际化水平，展现首府独特魅力。注重挖掘历史文化、地域风情和民族特色元素，精心打造达坂城镇、水西沟镇、长山子镇等一批特色小城镇。积极构建兵地协调机制，坚持优势互补，探索兵地融合发展新模式，提升兵地合作区发展水平，实现双赢、共同发展。

三是加强城市基础设施建设。加快推进轨道交通建设，力争地铁1号线北段年底试通车，5、6号线具备开工条件。启动西二环快速路、东进场路高架等工程，继续推进新医路西延、城北主干道机场高速互通立交等项目，建成跃进街东延、卫星路南延、天津路北延、喀什路东延二期。继续实施南部集中供热、天然气三期等工程，推进苏州路西延、卫星路、新医路西延等城市地下综合管廊建设。实施BRT延伸工程，建设公交专用道50公里。加快推进水源地和水库监控系统建设、河东再生水厂扩建及提标改造，启动“500”水库二期供水工程建设，确保城市供水安全。

四是提升城市精细化管理水平。大力实施市容市貌改善工程，推进卫生整治、立面改造和夜景亮化，抓好背街小巷改造和重点区域景观提升，展现干净美丽的城市形象。加快智慧城市建设，优化大数据平台功能，推动市政设施智能化感知和公共服务智能化共享。加快公交都市建设，推进交通智能化精细化管理，实施快速公交信号优先、公交智能化提升改造和公交线网优化调整。按照现代化国际城市建设标准，创新城市管理服务体系，推进行政综合执法规范化。增强城市建设管理透明度，完善社会参与机制，实现全民共治共管、共建共享。

五是全力推进大气污染治理。坚决落实治污、减煤、降尘、增电、改气“组合拳”，深入推进大气污染联防联控，确保全年空气质量优良天数达到250天以上。现有电厂实行“夏限产、冬减半”，逐步关停小电厂，最大限度控制原煤使用。继续加大城市天然气输配管网、场站和调峰储备设施建设，实施燃气锅炉烟气余热回收改造1000蒸吨，实施既有建筑供热计量及节能改造80万平方米。加快“电化”“气化”城市进程，实施750千伏输变电工程、风电光伏等清洁能源供暖示范项目和电动汽车充电基础设施建设，新增电采暖供热面积50万平方米。加大重点行业、企业污染治理力度，狠抓建筑工地、散装物料运输扬尘污染，强化机动车尾气治理。推进米东区、甘泉堡等重点区域综合治理，规划建设城市通风廊道，持续改善环境空气质量。

六是营造绿色宜居城市环境。围绕创建国家生态园林城市目标，按照“三区、四线”要求，守住生态资源底线，争取将我市打造成为“双城、双修”推广城市。全面提升城市生态绿化水平，加快东绕城生态圈和城北防护林绿带建设，新建5个荒山绿化改造提升公园，新增绿化面积1.12万亩，完成退耕还林1.76万亩，森林抚育1万亩，新建小游园及街旁绿地100个。完成水磨河景观改造，加快雅玛里克山绿化及景观大道建设，启动城北水景绿化项目建设。加强城市周边生态治理修复，做好矿山地质环境保护与恢复治理工作，加大森林和草原保护力度，加快柴窝堡湖国家级湿地公园建设，抓好一号冰川、十七户湿地、白鸟湖、头屯河谷、北部沙漠等生态环境综合治理。加强水资源保护和节约利用，严守水资源“三条红线”，扩大再生水推广使用规模。加强土壤污染防控，严格落实耕地保护制度，推动城市土地集约高效利用。加快发展循环经济，推广清洁能源和新能源汽车，促进绿色建筑及建筑产业化发展，新增装配式建筑70万平方米。建立项目准入负面清单，严格控制污染项目落地。健全生态环境监管和政绩考核制度，实行生态环境损害责任终身追究制和环境保护“一票否决”，确保生态环境安全。

六、进一步加强政府自身建设，提高服务能力和水平

为人民服务，对人民负责，受人民监督，让人民满意，是政府工作必须始终坚守的信念。面向打造“六个首府”和建设现代化国际城市的新征程，面对各族群众对美好生活的新期盼，我们要进一步加强政府自身建设，努力建设人民满意的政府。

一是坚持党的领导，践行为民服务宗旨。坚持党的领导是做好政府工作的根本保证。严格落实党内政治生活准则，严守政治纪律、政治规矩，始终坚持在党的领导下开展政府各项工作，以强烈的政治意识、大局意识、核心意识、看齐意识，不忘初心，奋力前行，确保党中央、自治区党委和市委的决策部署不折不扣贯彻落实。牢固树立全心全意为人民服务的宗旨，切实把以人民为中心的发展思想贯穿政府工作始终，体现在政府工作的方方面面，以实际行动和满腔为民情怀，实现好、维护好、发展好最广大人民的根本利益。

二是坚持依法行政，自觉接受监督。严格遵守宪法和法律，自觉运用法治思维和法治方式维护稳定、推动发展，坚持法定职责必须为，法无授权不可为，推进法治首府、法治政府、法治社会一体建设，进一步提高政府工作法治化水平。依法接受人大及其常委会的监督，自觉接受人民政协的民主监督，及时办理人大代表议案、政协提案。主动接受社会舆论和人民群众监督，保障群众知情权、参与权和监督权。坚持科学决策、民主决策、依法决策。扎实推进“七五”普法和依法治理工作。加快覆盖城乡居民的公共法律服务体系建设，拓展法律援助服务范围。健全依法维权和纠纷调解化解联动工作机制。深入推进政务公开，让权力在阳光下运行。

三是坚持强化纪律，切实转变作风。认真开展“学讲话、转作风、促落实”专项活动，聚焦总目标，树立问题导向，坚决果敢地向“四风”“四气”开刀，及时发现、坚决惩处“两面派”“两 面 人”，着力解决总目标认识不到位、从严治党有缺失、各项政策棚架、干部作风漂浮、失职渎职、基层基础薄弱、群众工作不到位、麻痹松懈厌战情绪等方面问题，推动广大干部作风有根本性转变，让“做老实人、说老实话、干老实事”成为各级干部干事创业的主旋律，着力打造忠诚干净担当的干部队伍。

四是坚持“严”字当头，狠抓廉洁政府建设。加强党风廉政教育，巩固深化党的群众路线教育实践活动、“三严三实”专题教育和“两学一做”学习教育成果，锲而不舍抓好中央八项规定和自治区党委、市委十条规定落实。坚持过紧日子，严格控制“三公”经费，进一步降低行政成本。加强行政监察、执法监察、效能监察，推进审计监督全覆盖，坚决纠正侵害群众利益的不正之风，积极营造风清气正的政治生态。牢固树立红线底线意识，坚定不移推进反腐倡廉工作，健全惩治和预防腐败体系，加大对权力运行的制约和监督，以零容忍的态度惩治腐败。

五是坚持强化责任，狠抓工作落实。牢固树立责任意识，始终做到守土有责、守土负责、守土尽责，自觉把岗位当阵地守、把工作当事业干、把奉献当本分，以强烈的事业心、责任心、使命感，全力完成党和人民交付的任务。坚持深入基层、深入实际，求真务实、开拓创新、真抓实干，一项一项工作地推进，一个环节一个环节地抓牢，一个问题一个问题地解决，一件事情一件事情地办好，坚决防止工作棚架、浮在面上，确保党中央、自治区党委和市委决策部署落地生根，各项惠民政策开花结果。

各位代表，实现社会稳定和长治久安总目标，是党中央赋予我们的神圣责任，是时代赋予我们的光荣使命，是首府各族市民的热切期盼。展望未来，我们重任在肩、信心满怀。让我们更加紧密地团结在以习近平同志为核心的党中央周围，在自治区党委、自治区人民政府和市委的坚强领导下，奋勇拼搏、团结奋进，为把首府建成西北地区特色鲜明、绿色健康、宜居和谐的现代化国际城市而不懈奋斗，以优异成绩迎接党的十九大胜利召开！
